# Effects of transcranial alternating current stimulation over right-DLPFC on vigilance tasks depend on the arousal level

**DOI:** 10.1038/s41598-021-04607-8

**Published:** 2022-01-11

**Authors:** Víctor Martínez-Pérez, Miriam Tortajada, Lucía B. Palmero, Guillermo Campoy, Luis J. Fuentes

**Affiliations:** Facultad de Psicología, Universidad de Murcia, Campus de Espinardo, 30100 Murcia, Spain

**Keywords:** Psychology, Human behaviour

## Abstract

Current theoretical accounts on the oscillatory nature of sustained attention predict that entrainment via transcranial alternating current stimulation (tACS) at alpha and theta frequencies on specific areas of the prefrontal cortex could prevent the drops in vigilance across time-on-task. Nonetheless, most previous studies have neglected both the fact that vigilance comprises two dissociable components (i.e., arousal and executive vigilance) and the potential role of differences in arousal levels. We examined the effects of theta- and alpha-tACS over the right dorsolateral prefrontal cortex in both components of vigilance and in participants who differed in arousal level according to their chronotype and time of testing. Intermediate-types performed the vigilance tasks when their arousal level was optimal, whereas evening-types performed the vigilance tasks when their arousal levels were non-optimal. Both theta- and alpha-tACS improved arousal vigilance in the psychomotor vigilance task (PVT), whereas alpha-tACS, but not theta-tACS, improved executive vigilance in the sustained attention to response task (SART), and counteracted the typical vigilance decrement usually observed in this task. Importantly, these stimulation effects were only found when arousal was low (i.e., with evening-types performing the tasks at their non-optimal time of day). The results support the multicomponent view of vigilance, the relevance of heeding individual differences in arousal, and the role of alpha oscillations as a long-range cortical scale synchronization mechanism that compensates the decrements in performance as a function of time-on-task by exerting and maintaining cognitive control attributed to activation of the right dorsolateral prefrontal cortex.

## Introduction

A variety of jobs require workers to be vigilant for rather extended periods of time. This is the case of taxi drivers when travelling long distances, pilots in long-haul flights, or surgeons when performing an operation, among many other examples. The ability to maintain attention also plays a fundamental role in academic and clinical settings. Typically developing children usually maintain concentration on teachers’ lectures to understand and retain large amount of information, an ability that children diagnosed with attention deficit hyperactivity disorder or autism spectrum disorder find extremely challenging. Detecting infrequent events is another type of activity that also requires maintaining attention throughout the activity, as occurs, for instance, when traffic controllers check for violations of traffic rules under congestion conditions or when teachers check for spelling mistakes in pupils’ reports. A common phenomenon when the aforementioned real-life activities are simulated in laboratory is the so-called vigilance decrement, which entails an impairment in performance (i.e., longer RTs and/or lower accuracy) with time-on-task^1–3^. However, the concept of vigilance is not unitary, and several forms of sustained attention may be involved in different vigilance tasks. Also, of special relevance in social, educational, and clinical settings is whether such vigilance decrements can be counteracted, and whether the effectivity of improvement procedures will depend on individual differences in arousal baseline. Here we set out to address these important issues given the relevance that vigilant attention has in many spheres of our life.

## Components of vigilant attention

According to Posner’s neurocognitive approach to attention, the alerting network is involved in both transiently preparing the individual to perceive and/or respond to a forthcoming target, which is referred to as the phasic component of the network, and in achieving and maintaining an optimal level of activation for longer periods of time (sustained attention), which is referred to as the tonic component of the network^4,5^. A right lateralized cortical network including the anterior cingulate cortex, the dorsolateral prefrontal cortex (DLPFC) and the right inferior parietal lobe^6^ (see^7^, for a review) has been involved in the tonic component, also referred to as vigilance. Accordingly, time-related drops in vigilance have been shown to correlate with right frontoparietal deactivation^8^. Likewise, electrophysiological studies have further shown that time-on-task variations in vigilance have been linked to the amplitude of theta and alpha cortical oscillations in the frontoparietal network^9^.

Further dissociations in the tonic component of the alerting network have been recently observed when different vigilance tasks are to be performed^10^. When the task is rather monotonous and tedious, with scarce requirements of cognitive or motoric demands, a kind of arousal vigilance mechanism is activated to maintain an optimal arousal level, allowing faster responses to stimuli of the environment. A prototypical task of this kind is the psychomotor vigilance task (PVT)^11^, where participants are told to respond as quickly as possible once a randomly presented target (e.g., a colored point) is detected. However, when the task makes strong demands of cognitive processes, such as in resolving conflict, flexibly switching between tasks, or withholding respond to infrequent targets, a kind of executive vigilance is then recruited^10^. A prototypical task of this kind is the sustained attention to response task (SART)^12^, where participants are told to respond to a succession of stimuli but inhibit the response just when an infrequent and randomly occurring target (e.g., a specific digit) is presented.

Beside both time-on-task and type of task, vigilance is affected by two important factors that determine how efficiently people sustain attention in vigilance tasks, the individual differences in circadian rhythms (e.g., chronotype^13^) and the oscillatory nature of attention (e.g., cortical oscillations in the frontoparietal network^9^).

## Chronotype in vigilance tasks

In synchrony with external time, circadian rhythms, our endogenous biological clock, determine our physiological and behavioral processes. Levels of arousal tend to stabilize along daytime, when the circadian rhythm system compensates the sleep-regulation homeostatic system, which accumulates pression to sleep depending on the time an individual spent awake^14^. However, as people undergo shifts in circadian phase, they may differ in their peak times where their levels of arousal are high^14–16^ leading to different circadian phenotypes that are usually classified as chronotypes. Thus, chronotype refers to the time of day preferred by individuals to perform their daily life activities and to sleep, which may result in morning-, evening- or intermediate-types. Morning-types reach their optimal functioning early in the morning, evening-types reach it late in the evening, and intermediate-types (the more frequent chronotype) are characterized by not having a pronounced circadian preference^14^. Although there are some physiological indices to determine people’s preferences for performing daily activities and sleeping, questionnaires have proven to be suitable for classifying individuals according to their chronotype^17^.

Martínez-Pérez et al.^13^ showed that evening-types performed better at their optimal time of day compared with their non-optimal time of day in both a task involving arousal vigilance (the PVT) and a conflict task that required cognitive control (the flanker task). Morning-types, however, only showed this kind of synchrony effect in the PVT, and this effect was, moreover, smaller in comparison with that found with evening-types. These findings suggest that fluctuations in vigilance are more apparent in evening-type than in morning-type individuals (see also^18^).

## Performance modulation in vigilance tasks

Some electrophysiological studies have shown that time-on-task variations in sustained attention can be modulated by non-invasive brain stimulation (NIBS)^19–28^. A first set of studies used transcranial direct current stimulation (tDCS) protocols^19,23–27^. Luna et al.^23^, for example, found that 1.5 mA of anodal high-definition tDCS over either the posterior parietal cortex or the DLDFC mitigated the executive vigilance decrement across time-on-task, whereas stimulation did not modulate the arousal component of vigilance. A second set of studies used transcranial alternating current stimulation (tACS) protocols. This technique has been proven to boost cognitive performance by enhancing the transfer of information among anatomically and functionally connected brain areas, which improve cognitive processes when the current is applied at specific oscillatory frequencies that concur with the endogenous regional synchronization involved in such cognitive functions. Both theta and alpha activity within the frontoparietal control network have been associated with either an increase or a decrease of cognitive control which is thought to be crucial for vigilance^29–40^. Congruently, and in accordance with oscillatory models of sustained attention^20,28^, previous tACS studies on sustained attention stimulated at these two frequencies. For instance, Clayton et al.^20^ found that 2.0 mA of alpha-tACS (10 Hz) over occipitoparietal cortex prevented deterioration in two different vigilance tasks. The authors concluded that alpha oscillations promote top-down control processes and vigilance stability. Rostami et al.^28^ tested the effects of 1 mA theta-tACS (6 Hz) over the medial prefrontal cortex and found pre-post stimulation differences in both frontal-midline theta power and performance on a sustained attention task. To our knowledge, however, none of these previous studies have simultaneously considered the three key factors regarding sustained attention in vigilance tasks: the multicomponent nature of vigilance, the potential role of individual differences in arousal level at baseline, and the oscillatory nature of sustained attention. Luna et al.^23^ took into account the distinction between arousal and executive components of vigilance^10^, but they did not heed neither the individual differences in arousal levels at baseline nor the oscillatory nature of sustained attention. Clayton et al.^20^ and Rostami et al.^28^, for their part, considered the oscillatory nature of sustained attention, but they did not heed neither the different components of vigilance, nor individual differences in arousal baseline.

Recent studies have highlighted the relevance of individual differences when assessing different methods of cognitive enhancing^21^ (see^41,42^, for recent reviews). In some cases, maximal effectivity is expected when people’s cortical excitability is below an optimal value according to an inverted U-shaped distribution, whereas either no effect or adverse effects are expected when the level of cortical excitability is at either optimal levels or above (although see^43^, for a demonstration of tDCS effects over the motor cortex when the level of cortical excitability was at optimal levels according to chronotype). Thus, it seems that NIBS effects could be modulated by pre-existing cortical excitation and inhibition baseline depending on factors such as age, hormonal fluctuations, neurotransmitter levels, and importantly, circadian influences^41^. The closer an individual is to its theoretical optimal arousal level, the lower the gain from stimulation is expected to be. In contrast, at suboptimal arousal levels gains may become greater^42^. Although previous studies have not purposely controlled for the influence of these individual differences at baseline, reliable tDCS effects have been observed when participants were at a non-optimal level of arousal due to sleep deprivation^24,25^, aging^19^, or some pathological conditions^44^.

## The present study

In two experiments, we set out to modulate vigilance performance in two tasks, one thought to tap arousal vigilance (the PVT) and other thought to tap executive vigilance (the SART). We investigated whether the two types of vigilance are causally related to theta and alpha rhythms by applying HD-tACS to the DLPFC, in line with previous related studies that targeted that region of the frontoparietal network for stimulation (e.g., see^23,24^). Differences in arousal levels were addressed by considering participants' chronotype and time of testing. We tested intermediate-types at the time of day when their arousal level was deemed to be optimal (Experiment 1) and evening-types at the time of day when their arousal level was expected to be low (Experiment 2). We hypothesized that evening-types would benefit more than intermediate-types from applying theta/alpha HD-tACS when performing the vigilance tasks, due to the former having lower baseline level of arousal than the latter at the time of testing.

## Methods

### Participants

Three hundred and ten undergraduates from the University of Murcia completed (online) the reduced version of the Horne and Östberg’s Morningness-Eveningness Questionnaire (rMEQ) developed by Adan and Almirall^45^. The rMEQ consisted of five items, with total scores ranging from 4 (definitively evening-types) to 25 (definitively morning-types). From this initial sample, undergraduates classified as intermediate-types (rMEQ scores from 12 to 17) and evening-types (rMEQ scores from 4 to 11) were invited to participate in Experiments 1 and 2, respectively. We recruited 60 intermediate-types (46 females; *M* age = 20.07, *SD* = 2.57; *M* rMEQ score = 14.08) to participate in Experiment 1 and 39 evening-types (35 females; *M* age = 19.38, *SD* = 1.95 M rMEQ score = 9.38) to participate in Experiment 2. The difference between experiments in the number of participants was unintended and resulted from the lower number of evening-types in the initial sample, which mimics that of the general population^46^.

All participants reported normal or corrected-to-normal vision and no chronic medical conditions. They gave written informed consent and received course credits for their participation. This study was approved by the Ethics Committee of the University of Murcia and was conducted conformed with the ethical standards laid down in the 1964 Declaration of Helsinki.

### General procedure

Experiment 1 and 2 were ran in parallel as intermediate- and evening-type participants were recruited. All participants were asked not to drink coffee or other stimulants for at least two hours prior to the tests. Participants from Experiment 1 (intermediate-types) came to the laboratory at 10:00 AM, 11:30 AM or 1:00 PM, times of the day when arousal is supposed to be at an optimal level for non-extreme chronotypes. Participants from Experiment 2 (evening-types) were cited at 8:00 AM, when their arousal level was expected to be low. We chose a parallel instead of a crossover design to avoid the potential learning effects that could conceal tACS effects^22^. Thus, participants came to the laboratory only on one occasion. Once in the laboratory, they were interviewed about their stimulant intake (none of them reported consumption). Next, they were randomly assigned to one of the three tACS stimulation conditions (sham, alpha, and theta), with the restriction that the number of participants per condition was equalized every three participants. There were 20 and 13 participants per stimulation condition in Experiment 1 and 2, respectively. Then, while receiving 25 min online stimulation, they performed the PVT for 10 min and, right after, the SART for about 18 min. Finally, participants were asked to rate their sensations related to stimulation (itching, pain, heat, etc.) from 0 to 10 (stimulation groups did not differ in self-report sensations, *F*s < 1 in the two experiments). In total, participants remained in the laboratory for approximately 45 min.

### Behavioral tasks

The tasks were controlled by software written in E Prime^47^. Participants were seated at a viewing distance of approximately 60 cm from the center of a 22-inch computer screen (resolution: 1920 by 1080 pixels) and responded using a Chronos® response box (Psychology Software Tools).

In the PVT, participants had to press a button as fast as possible when a red circle appeared on the screen. Each trial began with a random blank interval between 2 and 10 s. Then a red circle of 50 pixels in diameter popped up in the center of the screen and participants had to press a button. Then, the screen went blank and a new trial began.

In the SART, digits from 1 to 9 appeared in the center of the screen and participants had to press a button in response to all digit except the digit 3. Digits appeared in different font sizes (18, 27, 36, 45, or 54 points) for 250 ms, followed by an 800-ms mask (a circle with a cross inside) and a 100-ms blank screen (presentation rate: one digit every 1150 ms). Digits were selected at random with the restriction that each digit appeared once every nine trials and that the same digit did not appear in two consecutive trials. Participants completed 900 trials preceded by 18 practice trials.

### Stimulation

**Figure 1:**
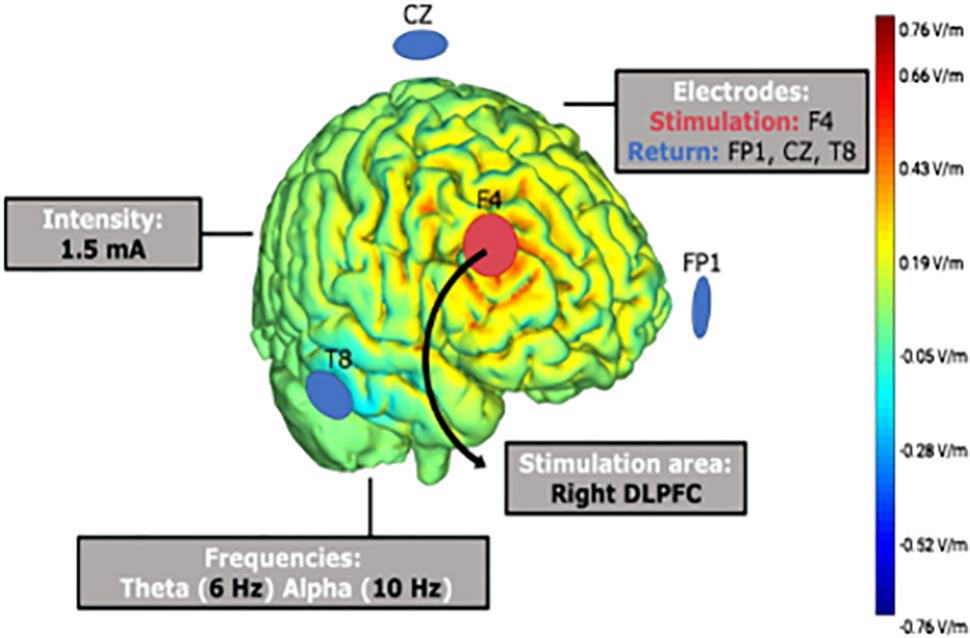
Brain stimulation technique. An illustration of the HD-tACS montage and the simulation of the electric field generated according to the StimWeaver software (Neuroelectrics).

Transcranial stimulation was delivered through a StarStim® wireless neurostimulator system (Neuroelectrics, Barcelona, Spain) connected to circular sponge-based electrodes (8 cm^2^), saturated with saline solutions to keep impedances below 10 kΩ. The target area of stimulation was the right DLPFC, located in F4 based on the 10–20 system. Three return electrodes were placed in a triangular scheme (T8, Cz, and Fp1), each of them with a 33% of current return (Fig. 1). Both experimenters and participants were blind to the stimulation conditions. During alpha- and theta-tACS, 1.5 mA intensity stimulation peak-to-peak was applied at 10 Hz and 6 Hz, respectively, right from the beginning of the PVT (including 30 s of ramp-up/down) and for 25 min. Sham-tACS stimulation was applied at 10 Hz (1.5 mA intensity) only at ramp periods to emulate the skin tingling sensation. It has been proven that this type of high-definition montages improve the focality of stimulation^48–50^.

### Statistical analyses

We analyzed the reaction times (RTs) on the PVT, the accuracy on the no-go trials of the SART (i.e., the proportion of no-go trials in which participants withheld their response), and the RTs on go trials of the SART. To detect possible changes in performance throughout the course of the tasks, we divided the PVT into five two-minute blocks, with between 12 and 23 trials per block (M = 18.69), and the SART into five blocks of 3.45 min, with 180 trials per block (160 go and 20 no-go). RTs were log-transformed to reduce the skewing in their distribution, and log-RTs beyond four times the semi-interquartile range from the median (0.36% and 0.59% of the data in the PVT and the SART, respectively, corresponding to 33 out of 9230 responses in the PVT and 448 out of 88,200 trials in the SART) were considered outliers and removed. The results were obtained by a series of 3 × 5 mixed analyses of variance (ANOVA), with stimulation (alpha, theta, sham) as the between-participants factor, and block (1–5) as the within-participants factor. The Greenhouse–Geisser sphericity correction was applied when necessary. Subsequent post-hoc tests used the Bonferroni method to correct for multiple comparisons. Additional independent *t*-tests were also conducted. We adopted a significance level of 0.05 for all analyses, which were performed with JASP 0.14^51^.

## Results

**Figure 2:**
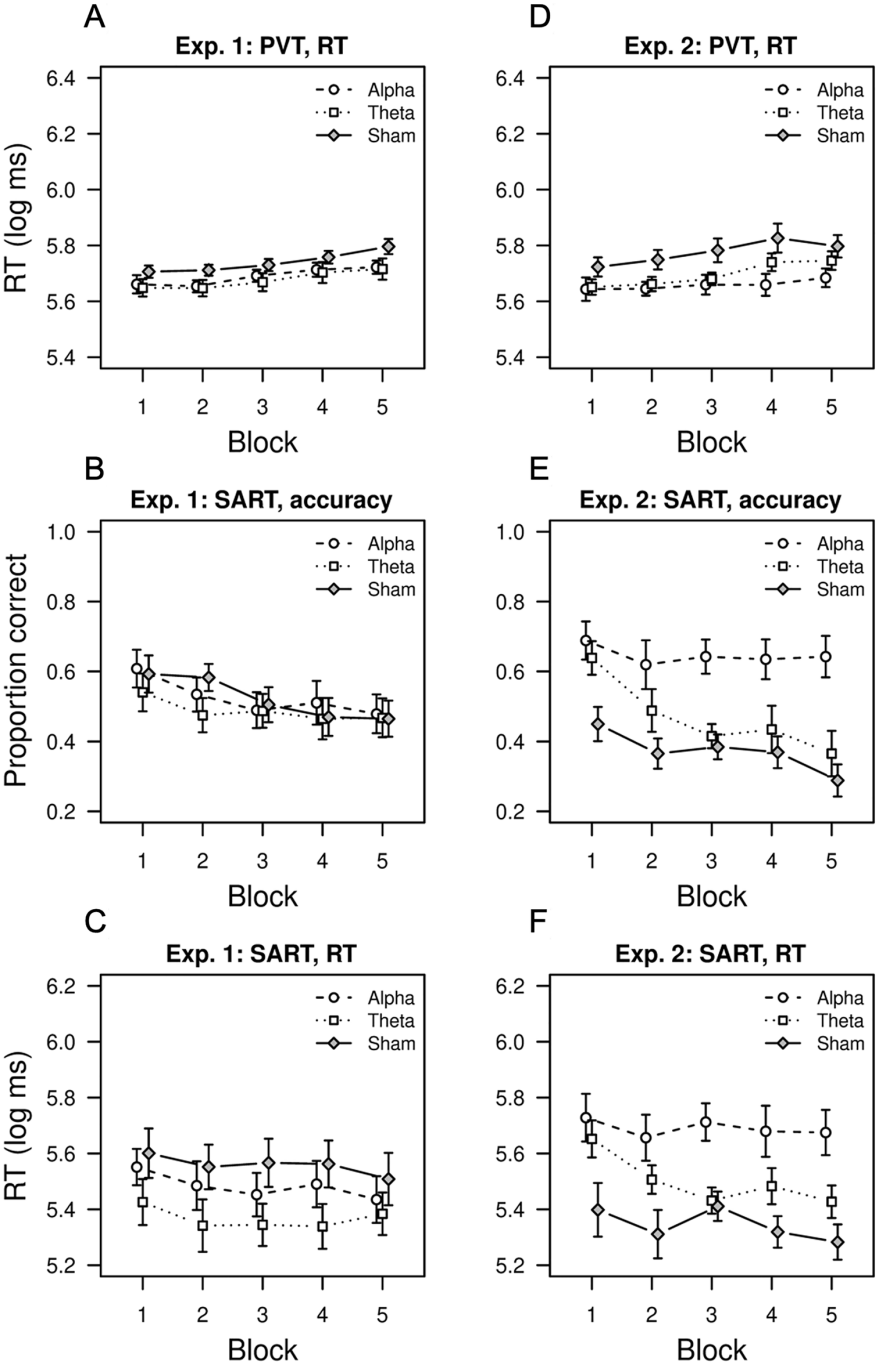
Results of Experiments 1 and 2. Arousal (PVT) and executive (SART) vigilance task performance as a function of tACS condition (alpha, theta or sham) across time-on-task. Intermediate-type participants performed Experiment 1 (panels **A**–**C**) while evening-type participants performed Experiment 2 (panels **D**–**F**).

Table 1 shows the statistics of the ANOVAs performed on the three variables considered (RT on the PVT, accuracy on the SART, and RT on the SART) for each experiment. Figure 2 depicts the mean RT and accuracy across experiments and conditions.

**Table 1:** Statistical results in Experiments 1 and 2 for the 3 (stimulation, S) × 5 (block, B) mixed ANOVAs on the RTs in the PVT, accuracy on no-go trials of the SART, and RTs on go trials of the SART.

Variable	Effect	Experiment 1				Experiment 2			
		F	df	p	η^2^_p_	F	df	p	η^2^_p_
PVT, RT	S	2.00	2, 56	.144	.067	3.50	2, 36	.041	.136
	B	13.39	2.3, 131.3	< .001	.193	10.70	2.7, 97.6	< .001	.229
	S × B	< 1	4.7, 131.3	.956	.009	1.42	5.4, 97.6	.220	.073
SART, accuracy	S	< 1	2, 56	.815	.007	10.38	2, 36	< .001	.366
	B	8.84	3.4, 189.1	< .001	.136	8.37	4, 144	< .001	.189
	S × B	1.01	6.8, 189.1	.423	.035	1.82	8, 144	.077	.092
SART, RT	S	1.53	2, 56	.226	0.052	8.11	2, 36	.001	.311
	B	3.60	3.3, 182.2	.012	0.061	4.13	2.7, 98.5	.010	.103
	S × B	< 1	6.5, 182.2	.626	0.026	1.41	5.5, 98.5	.223	.073

*S* stimulation, *B* block, the Greenhouse–Geisser sphericity correction was applied when necessary.

ANOVAs for Experiment 1 yielded statistically significant main effects of block, showing that performance in the two tasks declined as these tasks progressed. Post-hoc tests for the PVT revealed faster RTs in the first two blocks than in the last two blocks and faster RTs in the third block than in the last one, *t*s > 3.8, *p*s ≤ 0.002 (see Fig. 2 panel A). On the other hand, post-hoc tests for the SART revealed better accuracy in the first block than in the last three blocks and in the second block than in the last one, *t*s > 2.8, *p* ≤ 0.035, and slower RTs in the first block than in the third and the last one, *t* > 2.9, *p* ≤ 0.037 (see Fig. 2 panels B and C). Note that performance decline in the SART is revealed by faster rather than slower RTs (see the Discussion section for more details). Likewise, ANOVAs for Experiment 2 yielded main effects of block, showing performance deterioration throughout the tasks. Post-hoc tests revealed faster RTs in the first two blocks of the PVT than in the last two blocks, *t*s > 4.0, *p*s < 0.001 (see Fig. 2 panel D), better accuracy in the first block of the SART than in the other four blocks, *t*s > 3.5, *p*s ≤ 0.006, and slower RTs in the first block of the SART than in blocks 2, 4 and 5, *t*s > 2.8, *p*s ≤ 0.048 (see Fig. 2 panels E and F).

In contrast to Experiment 1, Experiment 2 yielded statistically significant main effects of stimulation. We first assessed whether there was a general effect of alpha/theta stimulation. As expected from previous findings, the stimulation conditions produced better performance than the sham condition in the PVT (i.e., shorter RTs, *t* = -2.51, *p* = 0.017) and the SART (i.e., better accuracy on no-go trials, *t* = 3.51, *p* = 0.001; and longer RTs on go trials, *t* = 3.37, *p* = 0.002). We then assessed whether there was any difference between the two stimulation conditions. As expected, we did not find any difference between alpha and theta conditions in the PVT (*t* < 1). However, in the SART, alpha stimulation produced significantly better accuracy on no-go trials (*t* = 2.9, *p* = 0.006) and longer RTs on go trials (*t* = 2.21, *p* = 0.033) than theta stimulation. In fact, theta stimulation did not differ from sham in both SART measures (*p*s > 0.05).

Regarding results of Experiment 2 with the SART, an inspection of Fig. 2 (panels E and F) suggests that the better performance in the alpha condition resulted from less or even no decrement in performance across block of trials. Accordingly, the stimulation by block interaction reached a marginal level of statistical significance (*p* = 0.077) although only for the accuracy analysis (see Table 1). Independent ANOVAs for each stimulation group revealed main effects of block for sham, *F*(4, 48) = 3.00, *p* = 0.027, *η*^2^_*p*_ = 0.200, and theta groups, *F*(4, 48) = 8.91, *p* < 0.001, *η*^2^_*p*_ = 0.426, but not for the alpha group, *F* < 1. Moreover, post-hoc tests comparing the first and the last block revealed significant accuracy decrements in theta and sham conditions, *t*s > 3.3, *p* ≤ 0.001, but not in the alpha condition, *t* < 1. To further support the differential pattern of performance across blocks of trials in the two stimulation groups (alpha and theta), a separate ANOVA including only the two stimulation groups confirmed that the stimulation by block interaction reached statistical significance, *F*(4, 96) = 2.973, *p* = 0.023, *η*^2^_*p*_ = 0.11. This pattern of results was also replicated with an alternative accuracy measure based on signal detection theory (the non-parametric index of sensitivity A)^52^.

Finally, we performed joint analyses with experiment (1, 2) as a factor to further assess how the effect of stimulation differed across experiments. We found no interaction involving experiment for the PVT. However, there was a stimulation by experiment interaction for accuracy and RT data in the SART, *F*(2, 92) = 4.05, *p* = 0.021, η^2^_p_ = 0.081, and *F*(2, 92) = 4.27, *p* = 0.017, *η*^2^_*p*_ = 0.085, respectively.

## Discussion

Sustained attention is crucial in many of our daily life activities, activities that have been simulated in the laboratory using vigilance tasks. However, the concept of vigilance is not unitary, and several components can be dissociated at both the behavioral and neural level^10,23^ (see^7^, for a review). In the present experiments, we have observed that both an arousal component, mainly involved in tedious and monotonous tasks (e.g., the PVT), and an executive component, mainly involved in vigilance tasks that require inhibitory control (e.g., the SART), can be differently modulated by non-invasive brain stimulation methods, as a function of individual differences in arousal baseline based on chronotype. When participants carry out tasks that require sustained attention in times of the day that, according to their chronotypes, match with their non-optimal level of arousal, performance is seriously affected in comparison to when they carry out the tasks in their optimal time of day^13^. Arousal baseline may be linked to the biological aspects of the circadian rhythms that, in interaction with the preference of individuals about when to perform their day life activities, generate variable levels of activation that affect their performance. Given that circadian influences affect cortical excitability^53–55^, we suggest that pre-existing excitation/inhibition baseline levels may determine whether brain stimulation will or will not have any effect on performance^41^. Accordingly, only evening-types, who performed the vigilance tasks at the non-optimal level of arousal (early in the morning) benefited from HD-tACS. These results agree with previous studies showing that different subgroups of participants with different baseline levels of cortical activation responded differentially to neuromodulation^21,56,57^.

People not only differ in arousal levels along the day, but also in their ability to sustain attention for extended periods of time. With time-on-task, participants usually show a progressive decrement in performance due to a decline in arousal levels that would affect their ability to sustain attention throughout the task. Due to the monotonous nature of some repetitive tasks that make scarce requirements of cognitive resources, it is expected that participants diminish their interest and lose the focus on the task. In previous research, we have observed that evening-type participants showed the synchrony effect, that is, they produced longer RTs when they performed the monotonous task (PVT) at their non-optimal time of day compared with when they performed the task at their optimal time of day^13^. Importantly, when the RT distribution was computed, the synchrony effect became larger at the slower end of the distribution, that is, when extreme fluctuations of attention emerged at the non-optimal time of day. These results suggest that sustained attention required in monotonous tasks fluctuates, mainly when the task must be carried out under low arousal conditions.

Here we show that entrainment at alpha and theta oscillations improved performance but only when the arousal component of vigilance was at non-optimal levels, that is, when evening-type participants carried out the task early in the morning. One plausible explanation is that for the arousal component of vigilance, brain stimulation has a general booster effect that shortens RTs, which would not be dependent on the concrete oscillation that has been entrained. A similar effect is even found when tDCS is applied over the prefrontal cortex under conditions of sleep deprivation^24^. Thus, it is activation of that particular brain area by NIBS techniques what seems to cause an increment in arousal vigilance.

A different pattern of results emerged when the task required strong demands of cognitive control like in the SART. It is noteworthy that, unlike other cognitive tasks, in which fast responses are associated with better performance, the opposite is true in the SART. Better performance is achieved when participants slow down their responses to nontarget digits on go trials to successfully refrain from responding to the infrequent target digit on no-go trials. Previous findings are consistent with this view (see^58–60^). In those studies errors on no-go trials were anticipated by an acceleration of response times on previous go trials. Our results showed that only stimulation at alpha oscillations improved executive vigilance performance. Alpha oscillations have been found to facilitate attentional stability^9,20^. Our findings fit well with such contention. By guiding attentional resources to the relevant stimulus (infrequent target digit), accuracy in withholding responses on no-go trials increased, fostering a high level of performance. In addition, alpha oscillations have been thought to exert an overall inhibitory effect on cortical processing, but also contribute to top-down inhibitory control mechanisms affecting task-irrelevant processes (see^9^, for review) that involve the prefrontal regions of the frontoparietal network^61^. Accordingly, alpha oscillations may have avoided participants from being affected by task-irrelevant thoughts that usually lead to mind-wandering states across time-on-task (see^62^). Note that a mind-wandering state here is interpreted as the opposite of a vigilance state^63^. As a consequence, vigilance decrement effects were not observed across time-on-task in the alpha stimulation group in comparison with theta stimulation and sham.

These effects on no-go trials accuracy may reflect a selective role of long-range synchrony effects of alpha oscillations in the activity of several brain areas that form part of the frontoparietal network, and hence with the cognitive operations supported by this network^38^. One relevant operation is concerned with phasic aspects of cognitive control, which can be triggered exogenously, for instance when an error has been committed^64^, and, of special relevance for the purpose of the present study, by salient target stimuli in a bottom-up manner. Bottom-up activation of cognitive control here may have been triggered by the sudden appearance of the infrequent target digit, which would activate the initiation of inhibitory control to withhold responding. Other relevant operation is concerned with the top-down maintenance (working memory) of task requirements, mainly involving the DLPFC^65,66^. Top-down maintenance of information in working memory is required here when participants are given the instructions of continuously responding to irrelevant frequent non-target stimuli and withholding responses just to the relevant infrequent digit 3.

These results support the model proposed by Clayton et al.^9^ by further providing it with causal evidence. This model predicts that performance in tasks that require sustained attention will be improved by entraining endogenous alpha and theta oscillations via tACS in frontal areas. Mechanistically, entrainment at both theta and alpha frequencies would promote changes in excitability of the frontoparietal circuit involved in vigilant attention^7^. The present results suggest that a more compelling model should heed the different components involved in vigilance tasks as well as the individual differences in arousal at baseline.

A remaining issue concerns the failure to observe any modulation of executive vigilance performance by applying theta-tACS. Fluctuations in cognitive control have been associated to power of theta band oscillations (e.g. see^67^), mainly in conflict tasks^9^. Thus, an effect of such slow oscillations should be expected mainly in the maintenance of task requirements in working memory, an operation that requires cognitive control. One possibility is that the typical theta oscillations at the local midfrontal region under full alertness condition, is no longer noticeable when alertness levels decrease, either because tasks have to be perform at non-optimal times of day, or because participants become drowsy^68^. Under such conditions, people are less capable of implementing cognitive control required by attentional demanding tasks. One way of counteracting the negative effects of low levels of arousal on maintaining cognitive control with time-on-task is to activate a reconfiguration of the cognitive control system via long-range cortical scale synchronization mechanisms between brain regions. The frequency band of such synchronization might depend on the kind of cognitive control required by the task at hand. In the case of conflict tasks, theta band oscillations seem to be the more appropriate^9,68^. In the case of tasks such as the SART, our current results suggest alpha band oscillations as the most appropriate. In any case, the result is that performance in such tasks is kept at the level of what is expected under conditions of full alertness. Thus, we claim that a main role of alpha oscillations at the long-range is to compensate the decrements in performance as a function of time-on-task by exerting and maintaining cognitive control attributed to the DLPFC. Future work using EEG and NIBS protocols will help determine how alpha-frequency oscillations communicate with the rest of the brain when people perform executive vigilance tasks under low arousal conditions.

A limitation of the present study is the lack of a balanced design that allowed us to test participants at both the non-optimal (low arousal) and optimal (high arousal) time of day according to their chronotype. However, despite the fact that no participants in either group performed the tasks in the evening, the evening-type participants tested in the morning (non-optimal time of the day) served to assess the effects of brain stimulation under low arousal levels, the main aim of the present study. In addition, the intermediate-type participants constituted a suitable control group because they performed the tasks at optimal times of day.

## Conclusions

To conclude, many daily activities require sustained attention for extended periods of time. For instance, in labor contexts, some jobs require working on nocturnal turns under sleep deprivation conditions, increasing fatigue and hence the risk of human error^69,70^. In education contexts, where classes are usually scheduled in the morning, students whose high level of arousal and cortical excitability occur in the morning hours (morning-types chronotype) would be in more favorable conditions than students with evening-type chronotype whose optimal level of arousal and cortical excitability occur in the evening, and vice versa when classes are scheduled in the evening^71^.

Recent studies have shown that NIBS methods pave the way to become an appropriate countermeasure to overcome the deleterious effect of vigilance decrements well beyond other common methods frequently used in different fields such as caffeine intake^24^ or the use of nootropic drugs such as modafinil^72^.

In the present study we went further to explore the benefits of brain stimulation in vigilance tasks by overcoming some shortcomings of previous studies. We dissociated two components of vigilance, one arousal component characteristic of monotonous tasks such as the PVT, and an executive component characteristic of cognitive demanding tasks such as the SART. Our results showed that tACS stimulation improved performance in the arousal component of vigilance. However, only stimulation at alpha frequency improved performance in the executive component of vigilance and counteracted the typical vigilance decrement usually observed across time-on-task. These results were observed only in evening-type participants tested when their levels of arousal were non-optimal, but not in intermediate-types participants tested when their level of arousal were optimal, highlighting the relevance of taking into account individual differences in pre-existing excitation/inhibition baseline levels in assessing cognitive enhancement.
